# Transition of Care from the Emergency Department to the Outpatient Setting: A Mixed-Methods Analysis

**DOI:** 10.5811/westjem.2017.9.35138

**Published:** 2018-02-08

**Authors:** Ashley C. Rider, Chad S. Kessler, Whitney W. Schwarz, Gillian R. Schmitz, Laura Oh, Michael D. Smith, Eric A. Gross, Hans House, Michael C. Wadman, Bruce M. Lo

**Affiliations:** * Highland Hospital, Alameda Health System, Department of Emergency Medicine, Oakland, California; † Durham VA Medical Center, Department of Emergency Medicine, Duke University, Durham, North Carolina; ‡ University of Texas at Austin Dell Medical School, Dell Children’s Medical Center of Central Texas, Department of Pediatric Emergency Medicine, Austin, Texas; § San Antonio Military Medical Center, Department of Emergency Medicine, San Antonio, Texas; ¶ Emory University, Department of Emergency Medicine, Atlanta, Georgia; || Ochsner Health System, Department of Emergency Medicine, New Orleans, Louisiana; # University of California Davis, Department of Emergency Medicine, Sacramento, California; ** University of Iowa, Department of Emergency Medicine, Iowa City, Iowa; †† University of Nebraska Medical Center, Department of Emergency Medicine, Omaha, Nebraska; ‡‡ Sentara Norfolk General Hospital/Eastern Virginia Medical School, Department of Emergency Medicine, Norfolk, Virginia

## Abstract

**Introduction:**

The goal of this study was to characterize current practices in the transition of care between the emergency department and primary care setting, with an emphasis on the use of the electronic medical record (EMR).

**Methods:**

Using literature review and modified Delphi technique, we created and tested a pilot survey to evaluate for face and content validity. The final survey was then administered face-to-face at eight different clinical sites across the country. A total of 52 emergency physicians (EP) and 49 primary care physicians (PCP) were surveyed and analyzed. We performed quantitative analysis using chi-square test. Two independent coders performed a qualitative analysis, classifying answers by pre-defined themes (inter-rater reliability > 80%). Participants’ answers could cross several pre-defined themes within a given question.

**Results:**

EPs were more likely to prefer telephone communication compared with PCPs (30/52 [57.7%] vs. 3/49 [6.1%] P < 0.0001), whereas PCPs were more likely to prefer using the EMR for discharge communication compared with EPs (33/49 [67.4%] vs. 13/52 [25%] p < 0.0001). EPs were more likely to report not needing to communicate with a PCP when a patient had a benign condition (23/52 [44.2%] vs. 2/49 [4.1%] p < 0.0001), but were more likely to communicate if the patient required urgent follow-up prior to discharge from the ED (33/52 [63.5%] vs. 20/49 [40.8%] p = 0.029). When discussing barriers to effective communication, 51/98 (52%) stated communication logistics, followed by 49/98 (50%) who reported setting/environmental constraints and 32/98 (32%) who stated EMR access was a significant barrier.

**Conclusion:**

Significant differences exist between EPs and PCPs in the transition of care process. EPs preferred telephone contact synchronous to the encounter whereas PCPs preferred using the EMR asynchronous to the encounter. Providers believe EP-to-PCP contact is important for improving patient care, but report varied expectations and multiple barriers to effective communication. This study highlights the need to optimize technology for an effective transition of care from the ED to the outpatient setting.

## INTRODUCTION

The vast majority of patients presenting to the emergency department (ED) are evaluated and subsequently discharged.^1^ Many of these visits will require a follow-up plan of care with a primary care physician (PCP). An appropriate transfer of information, within a reasonable time frame, must occur during this hand-off to ensure high-quality patient care and continuity of disease management.

### Background

Patient care transitions directly impact quality and patient safety. The discharge of a patient from the ED is a time of high vulnerability. Given the increasing complexity of medical care and the limitations that some patients may have due to language fluency or health literacy, the expectation of high-fidelity information transfer through discharge instructions alone is unrealistic in many cases.^2^ Prior studies have looked at handoffs between emergency physicians (EP) at shift change, between EPs and hospitalists, and between EPs and nursing homes.^3^–^6^ Common themes that arise from the literature regarding transitions of patient care from one healthcare provider to another are the need for bidirectional communication and a balance between standardization and flexibility.^2^

Both EPs and PCPs believe that coordination of care between the two settings is an important transition in healthcare.^7^ Communication between the EPs and PCPs has been regarded as unsatisfactory, if performed at all.^8^ Poor communication results in provider confusion regarding follow-up needs, which may predispose patients to error or adverse events.^9^–^10^ The process of hospital discharge to outpatient care currently has multiple barriers that contribute to poor transitions of care. These include unstructured communication systems, such as the electronic medical record (EMR), lack of longitudinal care and absence of follow-up standards.^11^ To begin to address barriers to effective transitions of care, it is necessary to investigate current systems of communication, with a focus on provider expectations and use of the EMR.

### Goals of this Investigation

The goal of this study was twofold. First, we aimed to characterize the current practices in the transition of care from the ED to the outpatient setting. Second, we sought to clarify providers’ preferences and use of EMR technology in managing that transition.

## METHODS

### Study Design

**Figure 1:**
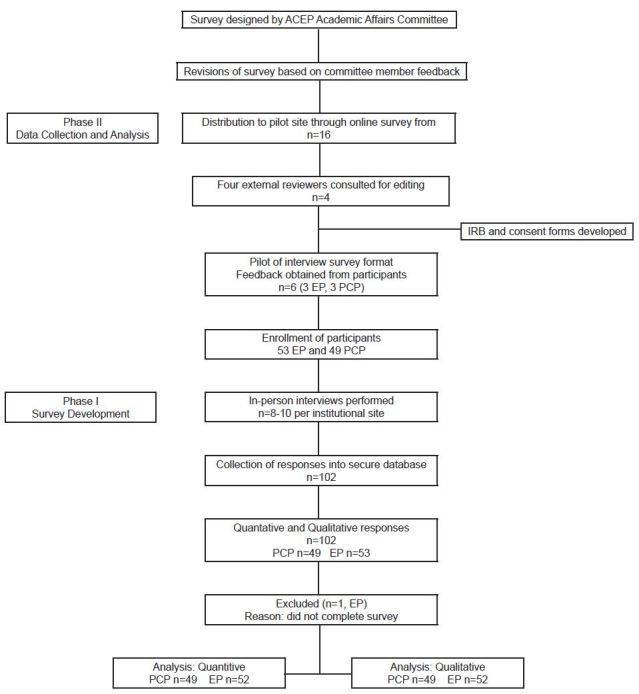
Consolidated Standards of Reporting Trials (CONSORT) flow diagram of responses to transition-of-care survey. *ACEP*, American College of Emergency Physicians; *EP*, emergency physician; *PCP*, primary care physician.

This was a prospective study using semi-structured interviews. We developed a mixed-methods survey based on literature review and modified Delphi technique.^12^ The survey was designed in two phases (Figure 1). In the first phase, the authors created the survey tool, which consisted of a set of general questions in regard to professional setting. The remainder of the survey was divided into questions specific for either EPs or PCPs. We included both multiple-choice questions and free-response questions to ensure capture of individual practices (Appendix). The survey was piloted via email to a group of 16 EPs and PCPs.

**Population Health Research Capsule**

What do we already know about this issue?

Transitions of care directly impact patient safety and healthcare quality. Discharge from the ED is a time of high vulnerability, yet there are few standards of communication in place.

What was the research question?

What are the current practices and preferences in the transition of care from the ED to the outpatient setting?

What was the major finding of the study?

Discrepancies exist between EP and PCP expectations and handoff preferences, and there are numerous barriers to communication.

How does this improve population health?

Standardized systems of communication should be the focus of improvements in the transition of care to the outpatient setting, with a specific focus on electronic medical record tools and technology.

After the initial pilot test, we reviewed and modified the survey for usability and redundancy. Additionally, we changed the format to an in-person interview. Four external reviewers were consulted and the survey was further refined for the in-person interview. Content validity and face validity were assessed by a process of multiple revisions based on pilot-test results, expert analysis, and triangulation.^13^ The survey was re-piloted in an in-person interview format with three EPs and three PCPs to obtain feedback on structure. We surveyed participants at eight different institutions. Participant anonymity was maintained by collection of data without identifying information.^14^, ^15^

### Setting

The survey tool was developed by members of the American College of Emergency Physicians (ACEP) Academic Affairs Committee. Academic and community physicians at eight different sites across the country participated in the study. We selected institutions based on author affiliation; these included University of California Davis, Eastern Virginia Medical School, Baylor College of Medicine, University of Nebraska, MetroHealth Medical Center, University of Iowa, Loyola University, and University of Texas San Antonio.

### Selection of Participants

We obtained institutional review board approval at each site. These institutions were primarily urban-based academic centers. Each author selected a convenience sample of EPs and PCPs. Only attending-level physicians were enrolled.

### Methods and Measurement

All participants underwent a verbal, informed consent prior to completing the survey. Interviews took place in person and lasted 15–20 minutes. We de-identified all data upon response submission. Data responses were collected electronically into a single electronic database at each site, and subsequently combined into a master database.

### Analyses

Quantitative data was extracted and entered into a processing program. We examined five demographic questions for all participants. The PCP survey contained 12 additional multiple-choice questions about transitions of care, and the EP survey contained eight additional questions. Data analysis was performed in SAS (version 9.2; SAS Institute, Inc. Cary, NC) using Fisher’s exact test. We performed qualitative data analysis for responses to the open-ended questions.

Themes were developed based on a grounded theory approach.^16^,^17^ Two independent coders used the constant comparative method to identify themes in the data collected from the interviews. If a discrepancy occurred, the reviewers discussed to achieve consensus. Themes were standardized for each question, and data were independently coded according to established themes. We assigned responses to one or more themes for each question. In an effort to minimize rater subjectivity regarding identified themes, we measured inter-rater reliability for each question with the goal of > 80% agreement.^18^ Percent agreement was based on the alignment of all selected themes for each question. We analyzed qualitative data for percent representation of individual themes. The number of responses for each theme was calculated and averaged between the coders for a percent representation of each theme.

## RESULTS

### Characteristics of Study Subjects

Between November 2014 and February 2015, 102 interviews were attempted. Forty-nine respondents were PCPs and 53 were EPs. Of these respondents, one EP participant did not complete the survey in its entirety. This respondent was omitted from the analysis, resulting in 101 responses analyzed.

PCPs were divided between family medicine, internal medicine, and pediatrics. All of the EPs were trained in emergency medicine, with one individual also trained in pediatrics. Both EPs and PCPs share similar demographic trends with the majority of the sites categorized as urban academic settings. The remainder of the demographic information is shown in Table 1.

### Quantitative Analysis

PCPs reported receiving communication about an ED visit much more frequently than EPs reported communicating back to the PCPs. Forty percent of PCPs reported actually receiving follow-up on their patients “most” of the time (81–100%), but a greater proportion (61.2%) felt that they should be contacted at this frequency. EPs preferred telephone communication to EMR and reported greater use of this modality, whereas PCPs preferred discharge communication through the EMR.

EPs were more likely to report no PCP communication for a patient with a benign and stable condition.

For patients requiring urgent follow-up, EPs were more likely to report the need for verbal communication prior to the patient’s discharge from the ED than PCPs. Both groups thought that a patient with an urgent condition required direct discharge communication with the PCP (Table 2).

Regarding perceptions of EMR use, EPs believe that the majority of PCPs (53.8%) use the same EMR and view the patient’s records directly. A minority of EPs believed that PCPs receive EMR notifications of a patient visit, whereas many PCPs reported receiving a notification.

### Qualitative analysis

**Figure 2:**
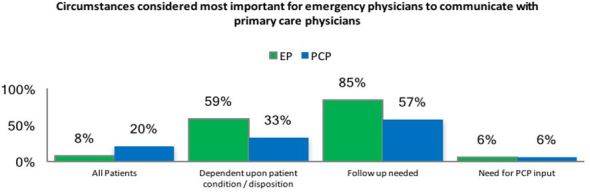
First qualitative question examining circumstances important for communication. *EP*, emergency physician; *PCP*, primary care physician.

Analysis of the 101 responses to qualitative-response questions identified thematic concepts for each question. The first question, “Under what circumstance is it important that the emergency physician communicate with a patient’s primary care physician?” demonstrated that follow-up needs were the most important reason to communicate with PCPs. Figure 2 demonstrates physician response.

**Figure 3:**
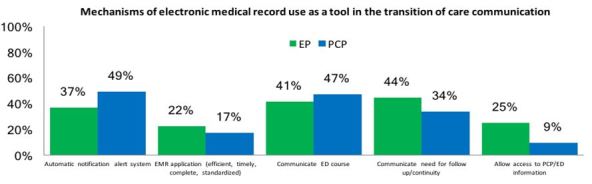
Second qualitative question regarding the use of the electronic medical record in the transition of care. *EMR*, electronic medical record; *EP*, emergency physician; *PCP*, primary care physician; *ED*, emergency department.

Physicians also responded to the question, “How should EMR be used as a tool in the transition of care?” More PCPs reported EMR notification/alert systems as a valuable use of EMR, compared to EPs who cited the EMR’s ability to aid in follow-up and continuity of care. Figure 3 demonstrates physician response.

**Figure 4:**
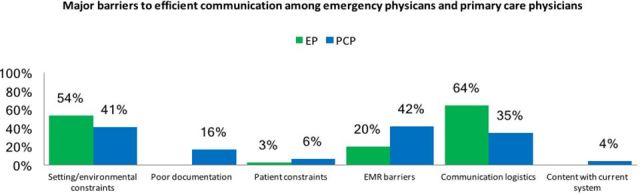
Qualitative question identifying major barriers to efficient communication. *EP*, emergency physician; *PCP*, primary care physician; *EMR*, electronic medical record Category descriptions: Setting and environmental constraints: high patient volume; coordinating time to call; and communication during non-business hours. EMR barriers: lack of EMR access or shared EMR; uncertain receipt of information; and limited EMR literacy. Barriers to communication: inability to identify the PCP; difficulty getting in touch with the appropriate provider; and lack of resources.

Finally, providers were asked, “What are major barriers to efficient communication with EP/PCPs?” Themes included setting/environmental constraints, communication logistics, and EMR barriers. Patient constraints were reported to a lesser extent by both groups. Poor documentation was mentioned among the PCP group. Figure 4 demonstrates physician response.

## DISCUSSION

Our multi-center prospective study examines and highlights the current practices and preferences for handoffs between EPs and PCPs and highlights quality gaps in the transition of the discharged ED patient back to the community. The study results suggest there is a discrepancy in provider expectations regarding best method of communication, and a disconnect between perception and reality of frequency of contact between ED and PCP providers. EPs preferred direct phone contact and communication synchronous to the encounter on patients needing urgent follow-up. In addition, EPs treated the communication of benign conditions differently that those with an urgent need. PCPs, on the other hand, preferred gathering information from the EMR and communication asynchronous to the encounter and wanted communication about non-urgent patients more often than EPs.

EPs may prefer to communicate by telephone because perhaps they are not aware of the extent to which PCPs automatically receive updates through the EMR. Less than half of EPs perceived that PCPs receive an EMR notification, while a majority of PCPs reported receiving an alert through the EMR. Since EMR notification was the preferred PCP method of communication, EPs might in future be more cognizant of the role of EMR notification to the PCP as a key component of transition of care for ED discharge.

There are also existing, under-used tools for communicating discharge information that are highly regarded and improve provider satisfaction.^19^ Limpahan et al. developed a set of best practices for patient discharge, including sending a summary to the PCP, performing medication reconciliation, and providing patient education.^20^ The authors suggest using the EMR as a potential avenue for automated inclusion of the described practices. Separate EMR systems have been identified as a challenge in the transition of care, while an interface for a shared EMR has been cited as a way to minimize transitions-of-care losses.^7^ Furthermore, the ability of EPs to provide an alert to the PCPs through flagging or email notification has been described as a potential tool for communication.

In the present study providers reported setting and environmental constraints including a high patient volume, coordinating time to call, and communication during non-business hours. A standard EMR notification system may alleviate some of these constraints; however, EMR barriers to effective transitions were also noted, including lack of EMR access or shared EMR, uncertain receipt of information, and limited EMR literacy. Other logistical barriers to communication included inability to identify the PCP, difficulty getting in touch with the appropriate provider, and lack of resources. These are systems issues that could be addressed with increased emphasis on the ED-to-outpatient communication. Specific strategies might include readily available electronic documentation of a patient’s PCP, shared EMR access among hospitals and clinics, and professional coordinators to relay information during the discharge process.

Healthcare providers believe that both technology and standardization should be the focus for future improvements in the transition of care.^8^, ^21^ Shared EMR access and EMR notifications are potential areas for development. There are also new tools of clinical communication that may bridge the gap between the synchronous phone communication preferred by EPs and the asynchronous EMR communication preferred by PCPs. Mobile health platforms that use HIPAA-compliant, secure text messaging can serve as an intermediate solution between phone message and EMR message, as these texts can satisfy the need for EPs to confirm delivery of an urgent message to a PCP, while allowing a small amount of asynchrony that does not disrupt the PCP’s workflow during a busy clinic day and is less intrusive than a phone call after hours. Further study is necessary to characterize the best structure and content of EMR notifications, in order to facilitate the transition of care from the ED to the outpatient setting.

## LIMITATIONS

There are several limitations to this study. Most notably, the participants comprised a convenience sample of physicians from eight academic institutions. All community physicians worked at a community site affiliated with one of the primary academic sites. The present study lacks representation from community sites without academic affiliation, military, and rural institutions. Our responses may not reflect practice patterns in these settings. This study also lacks input from mid-level providers and residents who are also involved in the hand-off process. Interviews were performed in-person and therefore may have led to reporting bias on the part of the participant.

Furthermore, this data is based on perception rather than objective measure of phone calls and EMR notifications, which is subject to recall bias. The qualitative questions served as a strategy to recruit more diverse responses. The process of coding synthesizes information, thus losing the context of specific statements in favor of categorizing data into themes. Finally, the majority of subjects in this study reported using EPIC EMR software. Other interfaces may allow for varying degrees of electronic communication between the ED and PCP, thus altering one’s perception of EMR utility.

## CONCLUSION

Our results highlight the need for a consistent system of communication, while also emphasizing the need for flexibility as EPs and PCPs work in distinct environments with different needs and expectations. Identifying these discrepancies is the first step in moving toward addressing them. EPs and PCPs should focus on working synergistically and view each other as partners working toward improved patient care. Future research should focus on new clinical communication tools for use between EPs and PCPs. Mobile health platforms or standardized, collaborative EMR tools have the potential to provide safer transitions back to the community.

## Supplementary Information



## Figures and Tables

**Table 1:** Demographic information of survey participants.

	PCP (N=49)	EP (N=52)
Specialty type		
Emergency medicine		51
Family medicine	25	
Internal medicine	15	
Pediatrics	8	
Combined emergency medicine/pediatrics		1
Combined internal medicine/pediatrics	1	
Years In practice		
Range	1–56	1–33
Average	15.3	10.4
Median	14	9.5
Type of practice		
Academic	34	36
Community	10	10
Both academic/community	4	5
No response	1	1
Setting		
Urban	21	27
Suburban	15	22
Rural	0	0
No response	13	3
Use of electronic medical record		
Yes	48	51
No	1	0
No response	0	1
Type of electronic medical record		
Epic	28	47
NextGen EHR	3	0
Allscripts	7	3
Sunrise	10	0
Azyxil	0	1
None	1	0
No response	0	1

*EP*, emergency physician; *PCP*, primary care physician.

**Table 2:** Results of quantitative analysis comparing primary care physician and emergency physicians responses to questions regarding direct discharge communication.

	PCP (N = 49)	EP (N = 52)	P value
PCP receives follow up after an ED visit “most of the time” (81–100%)	20 (40.8%)	0 (0%)	P<0.001
How ED visits are typically communicated to PCP			
EMR	36 (73.5%)	17 (32.7%)	P<0.001
Telephone	4 (9.2%)	36 (69.2%)	P<0.001
Preferred method of communication			
EMR	33 (67.4%)	13 (25%)	P<0.001
Telephone	3 (6.1%)	30 (57.7%)	P<0.001
Time frame for communication, benign condition prior to discharge			
Within 6 hours	2 (4.1%)	2 (3.8%)	P = 1
Within 24 hours	2 (4.1%)	3 (5.8%)	P = 1
Within 2 days	17 (34.7%)	12 (23.1%)	P = 0.271
Within 1 week	14 (28.6%)	6 (11.5%)	P = 0.045
Does not need	12 (24.5%)	7 (13.4%)	P = 0.205
Communication	2 (4.1%)	23 (44.2%)	P<0.001
Time frame for communication, urgent condition prior to discharge			
Within 6 hours	20 (40.8%)	33 (63.5%)	P = 0.029
Within 24 hours	10 (20.4%)	8 (15.4%)	P = 0.606
Within 2 days	15 (30.6%)	10 (19.2%)	P = 0.249
Within 1 week	2 (4.1%)	1 (1.9%)	P = 0.610
Does not need	0	0	
Communication	0	0	
PCP receives EMR notifications of ED visit	28 (57.1%)	11 (21.2%)	P<0.001

*EP*, emergency physician; *PCP*, primary care physician; *ED*, emergency department; *EMR*, electronic medical record
