# Supplementary material for: Transition of Care from the Emergency Department to the Outpatient Setting: A Mixed-Methods Analysis
Source: West J Emerg Med. 2018 Feb 8;19(2):245–53. doi: 10.5811/westjem.2017.9.35138 (PMC5851495; doi:10.5811/westjem.2017.9.35138)
Supplement: Supplementary file 1 [file wjem-19-245-s001.docx]

Appendix: Transition of Care Survey Instrument

Survey Begins:

Please answer the following questions regarding your practice in the outpatient or ED setting. On behalf of the ACEP Academic Affairs Committee, we thank you for your time.

Please give your specialty and provider type:

___Emergency Medicine

___Family Medicine

___Internal Medicine

___Pediatrics

___Nurse Practitioner

___Physician Assistant

___Combined EM/IM

___Combined EM/Pediatrics

Other: _______________________

Do you mostly work in the Emergency Department or in the primary care setting? *(Depending on what the subject checks, continue to section A or B)*

___Emergency Department Provider *(continue to section B)*

___Primary Care Provider *(continue to section A)*

Years in practice after completion of training: ____________________

Type of practice:

______Academic

______Community

______Military

______Other : _________________________

Do you use an EMR in your emergency department or office (PCPs)? ___Yes ___No

Name of EMR (if used) and associated hospital network: _________________________

**Please answer section A or B, based on your specialty:**

**Section A: For PCP providers**

1. I work in a (circle all that are true)

1. Integrated adult and pediatric outpatient clinic
2. Pediatric-only outpatient clinic
3. Adult-only outpatient clinic
4. Teaching clinic
5. Non-teaching clinic
6. Urban clinic
7. Suburban clinic
8. Rural clinic
9. In your opinion, do you receive adequate follow-up from ED providers when your patient is discharged from the Emergency Department?

Yes No

Please explain: __________________________________________

1. Please estimate the percent of patients for which you receive follow up from the emergency department provider after an ED visit:

a. <20%

b. 21%-40%

c. 41-60%

d. 61-80%

e. 81-100%

1. What percentage of time do you believe an ED provider **should** contact you? If you answered 100% of the time to the question above please disregard this question.

a. <20%

b. 21%-40%

c. 41-60%

d. 61-80%

e. 81-100%

1. How are ED visits **typically** communicated to you?

___telephone call

___email

___fax

___EMR notification (e.g. in-basket message)

___discharge summary

___I have shared EMR access and do not receive notification apart from the record

___I do not communicate with the ED provider

___Other: _______________________________________

Comments: __________________________________________

1. What is your **preferred** method of communication?

___telephone call

___email

___fax

___EMR notification (e.g. in-basket message)

___discharge summary

___My shared EMR access is sufficient

___I do not communicate with the ED provider

___Other: _______________________________________

Comments: __________________________________________

1. Under what circumstances is it important that the ED provider contact you? (For example: in all cases, discharged patients, admitted patients, patients in critical condition, only if I have seen the patient recently, etc). Please explain your response:

__________________________________________________________________

1. What is a desirable time frame for the receipt of information regarding a patient being discharged from the emergency department with a relatively benign diagnosis, in stable condition?

_____prior to discharge

_____within 6 hours of discharge

_____within 24 hours of discharge

_____within 2 days of discharge

_____within 1 week

______I do not need information on this type of patient

______other: ___________________________________

Comments:___________________________________________________________

1. What is a desirable time frame for the receipt of information regarding a patient who is discharged, but in need of urgent follow up?

_____prior to discharge

_____within 6 hours of discharge

_____within 24 hours of discharge

_____within 2 days of discharge

_____within 1 week

______ I do not need information on this type of patient

______other: ___________________________________

Comments:___________________________________________________________

1. Do you admit patients to the hospital?

______Yes

______No

______sometimes (Explain: _______________________)

Comments:___________________________________________________________

1. If you utilize an EMR in your office, is it the same EMR as the local ED most frequently used by your patients?

If necessary, please elaborate in the comments section.

______Yes, I have EMR with direct access to entire ED record

______Yes, I have EMR, but I do not have direct access to entire ED record

______Yes, I have EMR but it is different from ED record

_______I do not utilize EMR (please skip questions 12 and 13)

Other _________________________________

Comments:___________________________________________________________

1. If you have access, what part of the medical record do you view?

If necessary, please elaborate in the comments section.

_____entire medical record

_____ED note

_____transition of care note

_____discharge summary

_____other: _______________________________________

_____I do not have access

Comments: ___________________________________________________________

1. Does your office EMR have an alert system for receipt of information when a patient is discharged from the ED?

_______No alert system

_______email notification

_______alert within EMR

_______automated telephone call or text

_______Other: ___________________________

1. In your opinion, how should EMR be utilized as a tool in the transition of care?
2. In your opinion, what are some characteristics of a POOR hand-off in the transition of care from the ED back to your practice?
3. In your opinion, what are some characteristics of a GOOD hand-off in the transition of care from the ED back to your practice? What critical information should it include?
4. In your opinion, why is it important that the ED provider contact the PCP?
5. What are the major barriers to efficient communication with ED providers?
6. Additional comments: ___________________________________________________________

**Section B : For ED providers**

1. I work in a (circle all that are true)

a. Integrated adult and pediatric ED

b. Pediatric-only ED

c. Adult-only ED

d. Teaching hospital

e. Non-teaching hospital

f. Urban hospital

e. Suburban hospital

f. Rural hospital

2. What percentage of the time do you, the ED provider, personally communicate with the PCP provider for patients discharged from the ED?

a. <20%

b. 21%-40%

c. 41-60%

d. 61-80%

e. 81-100%

3. How do you **typically** communicate with a PCP provider?

___telephone call

___email

___fax

___EMR notification (e.g. in-basket message)

___discharge summary

___Most PCPs have shared EMR access and do not receive notification apart from the record

___I do not communicate with the PCP provider

___Other: _______________________________________

Comments: __________________________________________

4. What is your **preferred** method of communication with the PCP?

___telephone call

___email

___fax

___EMR notification (e.g. in-basket message)

___discharge summary

___Most PCPs have shared EMR access and do not receive notification apart from the record

___I do not communicate with the PCP provider

___Other: _______________________________________

Comments: __________________________________________

1. Under what circumstance is it important to communicate with a patient’s PCP? (For example: in all cases, discharged patients, admitted patients, patients in critical condition, etc). Please explain your response:

________________________________________________________________________

6. What is an acceptable time frame for communication with the PCP regarding a patient being discharged from the emergency department with a relatively benign diagnosis, in stable condition?

______prior to discharge

______within 6 hours of discharge

______within 24 hours of discharge

______within 2 days of discharge

______within 1 week

______ This type of patient does not require communication with the PCP

______other: ___________________________________

Comments:___________________________________________________________

7. What is an acceptable time frame for communication with the PCP regarding a patient that is discharged, but in need of urgent follow up?

_____prior to discharge

_____within 6 hours of discharge

_____within 24 hours of discharge

_____within 2 days of discharge

_____within 1 week

______This type of patient does not require communication with the PCP

______other: ___________________________________

Comments:___________________________________________________________

8. Do you utilize EMR to communicate with the PCP? If necessary, please elaborate on your response in the comments section.

______most PCPs utilize the same EMR and can directly view patient records

______most PCPs have access to shared EMR

______ most PCPs can view (only) the discharge summary

______ most PCPs receive an EMR notification when the patient was seen in the ED

______I do not utilize EMR to communicate with the PCP

______I do not know

______other : _________________________________________

Comments: ________________________________________________________________________

9. If your EMR automatically provides direct notification to the PCP for patients in the ED, how often do you speak to the PCP (e.g. telephonic, email) **in addition** to the EMR transfer of information? If there is no automatic notification, please select “not applicable.”

_____ 100% of the time

______>75% of the time

______50% of the time

______<25% of the time

______ only in select cases (please elaborate): _______________

______I do not communicate with the PCP aside from EMR

______not applicable

10. In your opinion, how should EMR be utilized as a tool in the transition of care?

11. In your experience, what are some characteristics of a poor hand-off in the transition of care communication?

12. For a transition that you believe went well, please describe what made it a GOOD handoff. What critical information should it include?

14. In your opinion, why is it important to contact a patient’s PCP?

13. What are the major barriers to efficient communication with PCPs?

14. Additional comments: ___________________________________________________________

Thank you for your participation.
